# Hybrid lineages of CD4^+^ T cells: a handbook update

**DOI:** 10.3389/fimmu.2024.1344078

**Published:** 2024-01-19

**Authors:** Antonio Bensussen, José Antonio Torres-Magallanes, Elena R. Álvarez-Buylla, Elena Roces de Álvarez-Buylla

**Affiliations:** ^1^Laboratorio de Neuroendocrinología, Centro Universitario de Investigaciones Biomédicas, Universidad de Colima, Colima, Mexico; ^2^Laboratorio de Genética Molecular, Epigenética, Desarrollo y Evolución de Plantas, Instituto de Ecología, Universidad Nacional Autónoma de México, Ciudad de México, Mexico; ^3^Centro de Ciencias de la Complejidad (C3), Universidad Nacional Autónoma de México, Ciudad de México, Mexico

**Keywords:** CD4+ T cells, hybrid lineages, plasticity, differentiation, immune response

## Abstract

CD4^+^ T lymphocytes have been classified into several lineages, according to their gene expression profiles and their effector responses. Interestingly, recent evidence is showing that many lineages could yield hybrid phenotypes with unique properties and functions. It has been reported that such hybrid lineages might underlie pathologies or may function as effector cells with protection capacities against molecular threats. In this work, we reviewed the characteristics of the hybrid lineages reported in the literature, in order to identify the expression profiles that characterize them and the markers that could be used to identify them. We also review the differentiation cues that elicit their hybrid origin and what is known about their physiological roles.

## Introduction

**Figure 1:**
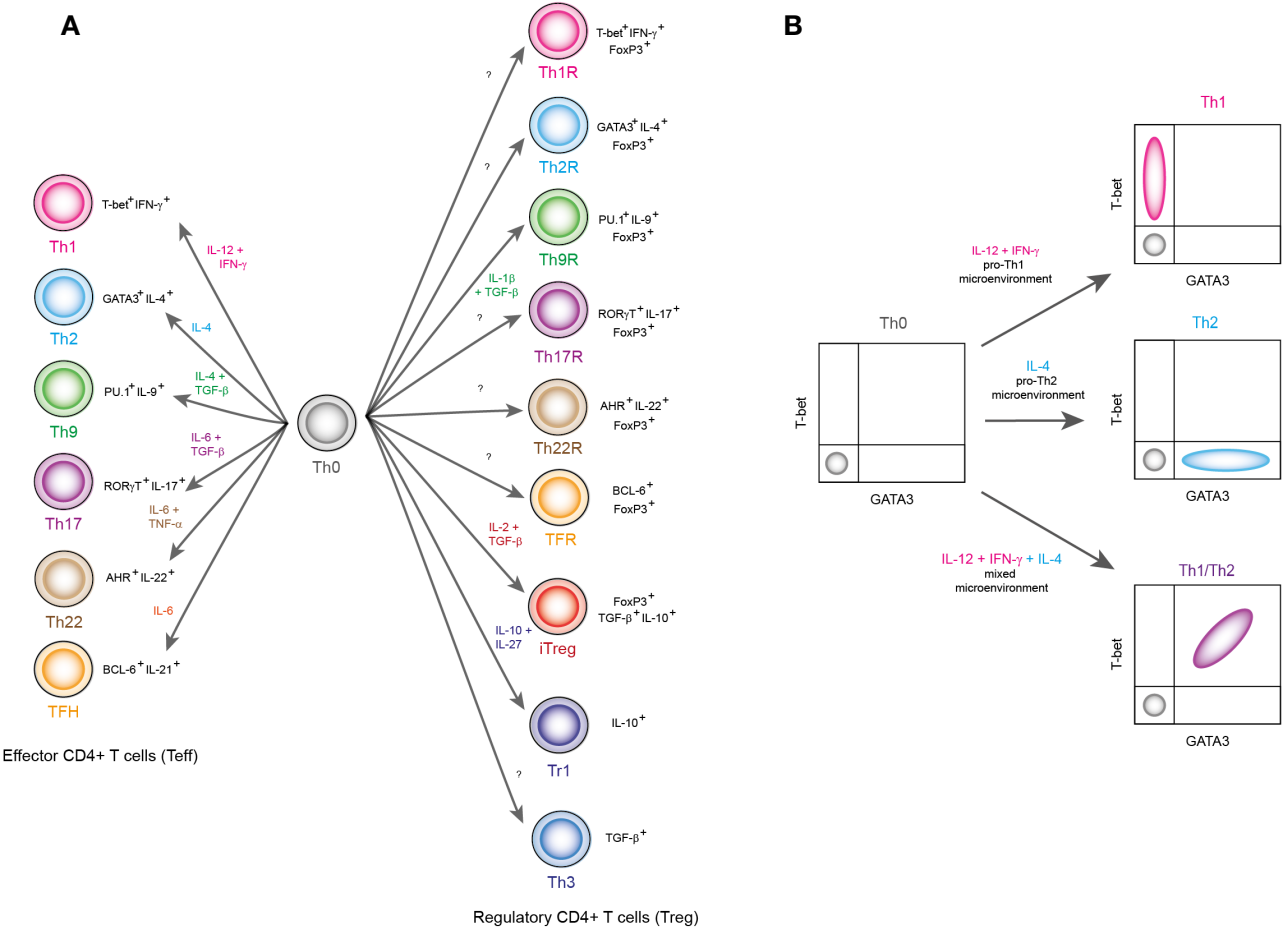
Classification of CD4^+^ T lymphocyte subpopulations. **(A)** Lymphocyte subpopulations are classified based on the transcriptional factors and cytokines they express. In this panel, differentiation environment is reported with colored labels as well as the name of each lineage. Also, the signature transcription factors and cytokines are shown in black labels. **(B)** This figure shows a schematic representation of flow cytometry results that show the emergence of hybrid lineages, which are outside the pure definitions of classical phenotypes Th1 and Th2. Here, the combination of the cytokines IFN-γ, IL-12 and IL-4 produces a population of T-bet^+^ GATA3^+^ hybrid cells, which correspond to a Th1/Th2 hybrid lineage.

Helper T lymphocytes (Th), also known as CD4^+^ T cells, are considered fundamental components of the immune system. In response to antigen presenting cells (APC), CD4^+^ T lymphocytes secrete a set of specific cytokines to coordinate immune responses. The cytokine signature is sustained by specific gene expression programs, that allows CD4^+^ T cells to be classified in particular subsets, that, in turn underlie their responses to certain types of threats and determined physiological functions (1). Two main CD4^+^ T cells groups have been distinguished: the regulatory cells (Treg) and the effector cells (Teff). Regarding to canonical effector lineages, they are divided in Th1, Th2, Th9, Th17, Th22 and TFH (T follicular lymphocytes) subsets, which can be identified as T-bet^+^ IFN-γ^+^ (Th1), GATA3^+^ IL-4^+^ (Th2), PU.1^+^ IL-9^+^ (Th9), RORγT^+^ IL-17^+^ (Th17), AHR^+^ IL-22^+^ (Th22) and BCL6^+^ IL-21^+^ (TFH) (**Figure 1A**). On the other hand, regulatory subsets are generally identified by expressing high levels of transcriptional factor FoxP3, as well as cytokines like TGF-β and IL-10 (**Figure 1A**). Each phenotype has a specific physiological role. For instance, Th1 is specialized in promoting an inflammatory response in response to viral infections, intracellular bacterial infections, neoplasms, or intracellular deregulations. In contrast, other subsets such as the iTreg downregulate these effector functions and are responsible for immune resolution and subsequent restoration of physiological homeostasis (2). It has been proposed that there are specific Treg lineages for every single CD4^+^ T effector subset (**Figure 1A**). The latter implies that all T helper cells can be specifically modulated (3).

As data accumulate, it has been shown that such a strict categorical approach is limited. For instance, when studying the ratio of Th1 (T-bet^+^) versus Th2 (GATA3^+^) cells under different conditions (4) (**Figure 1B**), there are scenarios in which both groups prevail, producing T-bet^+^ GATA3^+^ (Th1/Th2) double positive cells (**Figure 1B**). This hybrid subpopulation is able to maintain its phenotype in the form of memory cells and resist cell reprogramming (4), but it has been generally ignored because they have been found at low frequencies. Nevertheless, it is not clear whether these cells have a low populational frequency because in nature there are very few scenarios that elicit their differentiation, or they are rare because the conditions that are used in the laboratory are rare in nature. The differentiation processes that have been described under laboratory conditions are indeed relevant. But in order to understand the conditions that elicit hybrid lineages and their biological role, a wider range of physiological should be studied. Here, we review the physiological effects of CD4^+^ T cell hybrid lineages and what is currently known about them.

## Biological role of effector hybrid CD4^+^ T lineages

### Th1/Th2 lineage

These hybrid lymphocytes are characterized by being T-bet^+^ GATA3^+^ cells, as well as by expressing Th1 and Th2 cytokines. These cells were first characterized in mice infected by the helminths *S. mansoni* and *H. polygyrus* (4), but they also appeared in infections caused by the nematode *S. ratti*. These hybrids have been characterized in humans due to infections of the nematode *S. stercoralis* (5). Regarding its differentiation, Th1/Th2 hybrids appear due to the combination of IL-12, IL-4 and IFN-γ (4) (**Figure 1B**). In fact, they may present heterogeneous expression levels of T-bet and GATA3 due to variations on IFN-γ levels (4). Interestingly, these cells produce memory cells that are not susceptible to being reprogrammed after differentiation (4). Furthermore, in humans it was found that they present a predominantly Th1 profile (5) but in mice it is known that they maintain a balance between Th1 and Th2 (4). It is thought that at a physiological level, these cells act as immunomodulators without having to resort to immunosuppression, since they prevent the exacerbation of the Th2 response, which could potentially protect against allergies (4).

### Th1/Th9 lineage

This cell lineage can be identified as T-bet^+^ IL-9^+^ or alternatively as IFN-γ^+^ IL-9^+^, and it was first observed in humans as a byproduct of stimulation of Th17 cells with TGF-β (6). Years later, the Th1/Th9 hybrid was observed again in mice (7). Conventionally, it has been accepted that the way to induce Th9 polarization is using the combination of IL-4 plus TGF-β. However, when testing the combination IL-4 plus IL-1β, Th9 cells appeared that were T-bet^+^ IL-9^+^ (7). In addition, it was proven that they had strong antitumor activity when tested against the B16 melanoma cell line (7).

### Th1/Th17 lineage

This population of cells can be identified as T-bet^+^ RORγT^+^ or alternatively as IFN-γ^+^ IL-17A^+^. This lineage was first characterized in humans in the context of multiple sclerosis, and various studies have shown that it has a pathogenic effect (8). In addition, they have also been associated with severe orbitopathy of graves (9), type 1 diabetes, autoimmune uveitis and dry disease, intestine of Crohn’s disease, Sjogren’s disease, psoriasis (8) and cancer (10). Contradictorily, there are studies that suggest that these hybrids could have therapeutic effects. In mice, it has been reported that these cells present strong antitumor activity (11) and in humans, it has been seen that they promote recovery from sarcoidosis (12). Likewise, in observations in a murine model of colitis it was found that these hybrids can have a protective effect, since by hindering this hybrid population by deleting T-bet, the expression of IL-17 and IL-22 is triggered, drastically increasing the pathogenicity of inflammation of the intestine (13). Molecularly, Th1/Th17 hybrids are highly proliferative in response to stimulation by the TCR, they also increase their expression of GLUT1 and they can be stimulated by the action of IL-1β (8). Regarding their differentiation, in mice it was possible to obtain them in environments with IL-6, IL-23, IL-1β, IL-12 and low levels of TGF-β (11). *Ex vivo* observations show that in mice, IL-23 is essential to obtain these hybrids without affecting the differentiation of the Th1 and Th17 base phenotypes (13). On the other hand, in humans it is known that the secretion of IL-21 by central memory cells prevents the appearance of Th1/Th17 hybrids (13).

### Th1/Th22 lineage

This lineage is identifiable as T-bet^+^ AhR^+^ IL-22^+^, or alternatively as IFN-γ^+^ IL-22^+^. These cells were first found as a byproduct of murine Th22 lymphocyte differentiation in response to *C. rodentium* infection (14). Regarding their differentiation conditions, in mice it is known that Th22 cells obtained by the combination of cytokines TNF-α and IL-6 in an inflammatory context rich in IL-12, can transition to pure Th1 and Th1/Th22 hybrids (15). At the genetic level, experimental evidence suggests that this hybrid originates from a balance between the mutually exclusive regulation of AhR and T-bet (15), since the absence of any of these transcriptional factors hinders the expression of IL-22 (15). In humans, it has been found that this population appears before the worsening of multiple sclerosis begins (16). Furthermore, it was found that this cell lineage has autoreactive properties, and is insensitive to treatment with IFN-β, consolidating itself as a fundamental factor to hinder the treatment of multiple sclerosis (16).

### Th1/TFH lineage

Th1/TFH hybrids are characterized as T-bet^+^ BCL6^+^ CXCR5^+^, although they can also be labeled as T-bet^+^ IL-6^+^ or IFN-γ^+^ IL-21^+^ or IFN-γ^+^ BCL6^+^. These cells were characterized in mice infected with *P. chabaudi* (17, 18). Concerning to differentiation, these cells appear in environments rich in IFN-γ and IL-27, the latter being responsible for biasing differentiation towards the TFH side (17). Interestingly, it was found that in response to prolonged *P. chabaudi* infections, Th1/TFH hybrids tend to be biased towards the TFH side, increasing the stimulation of antibody production (17). However, brief infections bias differentiation towards the Th1 side, which favors the Th1/TFH memory being able to protect against reinfection by *P. chabaudi*. This suggests that the emergence of the Th1/TFH hybrid population could have beneficial effects in the short term, but may represent a subsequent risk (18). Additionally, these cells were found in human nasal polyps. In humans, this lineage appears through the action of IL-12; and these hybrids were found to be responsible for the inflammation of nasal polyps (19). Counterintuitively, it is suggested that the role of this Th1/TFH population is protective, since differentiation towards the TFH pole protects from damage produced by an enhanced Th1 response (19).

### Th2/Th9 lineage

Th2/Th9 hybrids identifiable as IL-4^+^ IL-9^+^ or alternatively GATA3^+^ PU.1^+^, were observed in patients as a product of mutations in STAT1 or STAT3. It is speculated that these hybrids could make patients prone to contracting candidiasis, cancer and sinopulmonary infection (20). Similarly, these hybrid cells were observed at low frequencies in *E. granulosus* infection (21). In mice, these hybrids were observed as a byproduct of papain-induced airway inflammation (22). However, the conditions of its differentiation and its exact physiological function are unknown.

### Th2/Th17 lineage

This hybrid population can be identified as GATA3^+^ RORγT^+^; or alternatively, as IL-4^+^ IL-17A^+^ or GATA3^+^ CD161^+^. Th2/Th17 hybrids were found in patients with asthma, and have the ability to induce IgE secretion (23). Interestingly, it was not possible to obtain these hybrids under *in vitro* conditions, but CCR6^+^ CD161^+^ memory cells produced these hybrids in IL-4-rich environments, suggesting that the Th2/Th17 population could appear in this context (23). Furthermore, this population was also found in a murine model of asthma, and it was found that they had the ability to exacerbate the inflammation produced in asthma (24). Years later it was found that in the context of asthma in mice, these hybrids could differentiate based on the activity of dendritic cells when repeatedly exposed to pro-inflammatory treatments with ovalbumin (25). Memory cells with Th2/Th17 phenotype were recently found, and they were linked to the appearance of Palmoplantar pustulosis (26). Regarding differentiation of these hybrids in mice, Th2/Th17 cells were detected in low quantities as byproducts of an unconventional pro-Th17 differentiation pathway, in which the combination of IL-9 plus TGF-β was used (27).

### Th9/Th17 lineage

These hybrids have been identified as IL-9^+^ IL-17^+^. Experimentally, it has been seen that Th9/Th17 hybrids appear due to the joint stimulation of the cytokines TGF-β, IL-1β, IL-6, IL-21 and IL-23 (6). Alternatively, it was also found that they could differentiate in the presence of the combination of cytokines TGF-β plus IL-12, or TGF-β plus IL-1β, or by the classic Th9 differentiation conditions, TGF-β plus IL-4 (6). Although, they have also appeared due to the combination of cytokines TGF-β, IL-4, IL-1β and IL-23 (28). This lineage was first characterized in patients with autoimmune diabetes; being significantly more frequent than what is observed in healthy people (6). Later these cells were found again in both humans and mice, in the context of autoimmune gastritis (28). However, trials showed that the decrease in the Th9/Th17 hybrid population severely worsened the development of the disease (28). Therefore, it is suggested that these cells could have a protective effect against damage caused by the Th17 response (28).

### Th9/TFH lineage

This hybrid line, defined by the presence of the IL-9^+^ BCL6^+^ markers, should ideally not exist. This is because Th9 differentiation conditions inhibit BCL6 activity, yet a tiny fraction of this hybrid lineage was found in mice (29). Experimental data suggest that IL-2 in the presence of a classical pro-Th9 environment could stabilize this hybrid population, albeit at low levels (29). It is not known if it has any specific physiological function.

### Th17/Th22 lineage

Th17/Th22 hybrids can be identified as IL-17^+^ IL-22^+^ or alternatively as RORγT^+^ BCL6^+^. Originally, it was thought that these cells were pathogenic, since in murine models of colitis, increasing their frequency triggers the severity of intestinal inflammation (13). However, recent evidence suggests that these cells play an important role in the regulation of intestinal homeostasis; since in rhesus macaques infected with simian immunodeficiency virus (SIV), decreasing the frequency of these hybrids, enhanced the severity of SIV infection (30). There are no reports on the conditions that promote their differentiation, but it was observed that the presence of IL-23 increases the differentiation of these hybrids (13). Additionally, it has been reported that the absence of T-bet drastically increases its amount (31).

## Biological role of regulatory hybrid CD4^+^ T lineages

### Th1/Th2-like regulatory lineage

Recently, experimental evidence was found for the existence of a hybrid Th1/Th2-like regulatory lineage in humans (32). This lineage was identified as T-bet^+^ GATA3^+^ FoxP3^+^, and its prevalence was observed to decrease in patients with cardiovascular disease (32). The differentiation conditions of this phenotype are unknown, and it is thought that it may have a protective effect against cardiovascular disease.

### Th1/Th17-like regulatory lineage

In humans, a regulatory hybrid Th1/Th17-like lineage was found; labeled as T-bet^+^ RORγT^+^ FoxP3^+^ (33). This lineage was found as part of memory cells and was seen to have a high proliferative capacity compared to other regulatory cells. At the systemic level, these hybrids were predominantly observed in the colon. Interestingly, in the context of cancer, a significant decrease in this cell population was observed (33). Furthermore, it was observed that in the context of cancer, Th2-like regulatory cells (GATA3^+^ FoxP3^+^) are attracted to the tumor, and inhibit regulatory Th1/Th17-like cells, so it is thought that they may have a protective role against the tumors (33). The conditions that favor their differentiation *in vitro* are unknown.

## Complexity of hybrid CD4^+^ T lineages

In addition to the limitations that hybrid lineages represent for the conventional classification system, there are effector cells whose classification is impossible. For example, effector cells were found in mice, whose phenotypes were IL-2^+^, TNF^+^, IL-2^+^ TNF^+^ IL- 22^+^ IL-5^+^, GM-CSF^+^ IL-5^+^ IL-13^+^ and IL-2^+^ IFN-γ^+^ TNF^+^ IL-22^+^ IL-5^+^ (34). In addition, it was also shown that even the traditionally defined lineages might present notable variations, for example, Th1 cells identified as IFN-γ^+^, and IFN-γ^+^ TNF^+^ GM-CSF^+^ IL-22^+^ have been described (34). Physiologically, these polyfunctional lymphocytes, i.e., CD4^+^ T cells that express more than one biologically active cytokine, have been found to have completely different functions from each other (35). Indeed, polyfunctional Th1 lymphocytes identified as IFN-γ^+^ TNF-α^+^, IL-2^+^ provide effective protection against COVID-19 (36), unlike conventional Th1 lymphocytes (IFN-γ^+^). Interestingly, Th17/Th22 hybrid cells were found to be polyfunctional in healthy *Rhesus* macaques, because they express up to four additional cytokines (30). In the presence of SIV, not only did the Th17/Th22 population decrease, but polyfunctional hybrid cells also decreased in frequency (30). Furthermore, increments of these cells have been found to be correlated with the reduction of SIV viremia levels, thus suggesting their role in health (30).

## Discussion

Traditionally, CD4^+^ T lymphocytes were thought to have clearly distinguishable phenotypes, with specific functions and regulatory pathways. Nowadays, several experimental results suggest the opposite. In fact, the evidence shows that the differentiation of CD4^+^ T lymphocytes occurs in the form of a gradient, which implies that hybrid subpopulations can be limitless (**Table 1**). The open question is to understand how such lineages emerge. The answer requires dealing with the complexity of the microenvironments in which the differentiation of CD4^+^ T cells occurs, including intracellular alterations such as epigenetic changes. In this sense, systems biology allows to visualize the effect of intrinsic and extrinsic variables that impact the differentiation of CD4^+^ T cells, in conditions that are not feasible to achieve experimentally. For example, by using multistable models, it has been possible to reproduce the molecular mechanisms that originate all differentiation patterns of the canonical lineages of CD4^+^ T lymphocytes (37). In addition, these models predicted that stochastic intracellular perturbations in the signaling and the expression of signature transcription factors caused the emergence of non-canonical lineages (37).

**Table 1:** Hybrid lineages of CD4^+^ T cells.

Phenotype	Markers	Differentiation signals	Source of observations
Th1/Th2	T-bet^+^ GATA3^+^, IFN-γ^+^ IL-4^+^	IL-12 + IL-4 + IFN-γ	*Mus musculus* (4), *Homo sapiens* (5)
Th1/Th9	T-bet^+^ IL-9^+^, IFN-γ^+^ IL-9^+^,	TGF-β + IL-4 + IL-1β	*Mus musculus* (7), *Homo sapiens* (6)
Th1/Th17	T-bet^+^ RORγT^+^, IFN-γ^+^ IL-17A^+^	IL-6 + IL-23 + IL-1β + IL-12 + TGF-β ^low^	*Mus musculus* (13), *Homo sapiens* (8)
Th1/Th22	T-bet^+^ AhR^+^ IL-22^+^, IFN-γ^+^ IL-22^+^	TNF-α + IL-6 + IL-12	*Mus musculus* (14), *Homo sapiens* (16)
Th1/TFH	T-bet^+^ BCL6^+^ CXCR5^+^, T-bet^+^ IL-6^+^, IFN-γ^+^ IL-21^+^, IFN-γ^+^ BCL6^+^.	IFN-γ + IL-27	*Mus musculus* (17), *Homo sapiens* (19)
Th2/Th9	IL-4^+^ IL-9^+^, GATA3^+^ PU.1^+^	Deletion of STAT1 or STAT3	*Mus musculus* (22), *Homo sapiens* (20)
Th2/Th17	GATA3^+^ RORγT^+^, IL-4^+^ IL-17A^+^, GATA3^+^ CD161^+^	IL-9 + TGF-β (only as a byproduct in mice)	*Mus musculus* (24), *Homo sapiens* (23)
Th9/Th17	IL-9^+^ IL-17^+^	TGF-β + IL-1β + IL-6 + IL-21 + IL-23, TGF-β + IL-4 + IL-1β + IL-23	*Mus musculus* (28), *Homo sapiens* (6)
Th9/TFH	IL-9^+^ BCL6^+^	TGF-β + IL-2 + IL-4	*Mus musculus* (29)
Th17/Th22	IL-17^+^ IL-22^+^, RORγT^+^ BCL6	IL-23 excess ()?	*Mus musculus* (13), *Rhesus macaques* (31)
Th1/Th2-like regulatory	T-bet^+^ GATA3^+^ FoxP3^+^	Unknown	*Homo sapiens* (32)
Th1/Th17-like regulatory	T-bet^+^ RORγT^+^ FoxP3^+^	Deletion of T-bet	*Homo sapiens* (33)

Moreover, continuous approaches of multistable models showed that the majority of Th phenotypes follow bimodal dynamics, but depending on the levels of polarizing cytokine, non-canonical phenotypes may emerge (38). Indeed, the model predicts that microenvironments mixed with IL-4^+^ and IFN-γ^+^ affect cell differentiation depending on their concentration. To be precise, high levels of exocrine IFN-γ or IL-4 produces either Th1 or Th2 cells that are not plastic, which means that even in presence of the contrary cytokine, they maintain their phenotype (38). On the contrary, low levels of exocrine IFN-γ or IL-4 generates plastic Th1 or Th2 phenotypes (38). Molecularly, the model predicts that Th2 lineage requires to produce autocrine IL-4 to maintain its phenotype. But if the autocrine production of this cytokine is reduced, then it may appear Th1/Th2 hybrids (38), which implies that plasticity is governed by the microenvironment and the intracellular state. Concerning to the effect of other external influences, multistable models reveled that, systemic hormones such as insulin (39) or local bioactive signals like adipokines (40) are able to bias CD4^+^ T cell differentiation towards inflammatory phenotypes, reducing the presence of regulatory lineages even in anti-inflammatory microenvironments.

Recently, independent studies have demonstrated these predictions. Indeed, inhibiting the enzyme ornithine decarboxylase increased epigenomic alterations in histone acetylation, which augments transcriptional noise as well as the frequency of hybrid lineages (41). Similarly, it has been reported that the majority of genes responsible for the emergence of Th1 and Th2, present bimodal dynamics, and only a fraction of them present mixed behaviors (42). Furthermore, it has been demonstrated that high exposition to polarizing cytokines IL-12 and IFN-γ during the infection of *P. chabaudi*, reduces the frequency of Th1/TFH hybrids (18). On the contrary, low exposition to such cytokines, increases the hybrid subset (18). Perhaps, these mechanisms could be extrapolated to other immune cells, like CD8^+^ T lymphocytes. In fact, Tc1/Tc17 lymphocytes (identified as IFN-γ^+^ IL-17^+^ CD8^+^) have been described as important downregulators of encephalitis (43). In conclusion, the study of hybrid lineages of CD4^+^ T lymphocytes is important to further understand the complex dynamics of immune systems and their plastic responses under various cues. Understanding such complexity will necessarily require the use of formal and computer modeling approaches.

## Author contributions

AB: Conceptualization, Data curation, Investigation, Visualization, Writing – original draft. JT-M: Conceptualization, Data curation, Investigation, Visualization, Writing – original draft. EÁ-B: Conceptualization, Funding acquisition, Project administration, Resources, Supervision, Writing – review & editing. EdA-B: Conceptualization, Funding acquisition, Project administration, Resources, Supervision, Writing – review & editing.
